# ZEB2 inhibits HBV transcription and replication by targeting its core promoter

**DOI:** 10.18632/oncotarget.7435

**Published:** 2016-02-17

**Authors:** Qiao He, Wanyu Li, Jihua Ren, Yecai Huang, Ying Huang, Qin Hu, Juan Chen, Weixian Chen

**Affiliations:** ^1^ Department of Laboratory Medicine, The Second Affiliated Hospital of Chongqing Medical University, Chongqing, China; ^2^ The Second Affiliated Hospital and the Key Laboratory of Molecular Biology of Infectious Diseases designated by the Chinese Ministry of Education, Chongqing Medical University, Chongqing, China; ^3^ Department of Radiation Oncology, Sichuan Cancer Hospital, Chengdu, China; ^4^ Department of Infectious Diseases, The Second Affiliated Hospital of Chongqing Medical University, Chongqing, China

**Keywords:** ZEB2, HBV replication, HBV core promoter

## Abstract

Hepatitis B virus (HBV) infection is a major cause of liver diseases, especially liver cirrhosis and hepatocellular carcinoma. However, the interaction between host and HBV has not been fully elucidated. ZEB2 is a Smad-interacting, multi-zinc finger protein that acts as a transcription factor or repressor for several signaling pathways. This study found that the expression of ZEB2 was decreased in HBV-expressing cells. Overexpression of ZEB2 inhibited HBV DNA replicative intermediates, 3.5kb mRNA, core protein level, and the secretion of HBsAg and HBeAg. In contrast, ZEB2 knockdown promoted HBV replication. Furthermore, ZEB2 could bind to HBV core promoter and inhibit its promoter activity. Mutation at the ZEB2 binding site in HBV core promoter eradicated ZEB2-mediated inhibition of HBV replication. This study identifies ZEB2 as a novel host restriction factor that inhibits HBV replication in hepatocytes. These data may shed light on development of new antiviral strategies.

## INTRODUCTION

HBV infection leads to a wide spectrum of liver diseases, including acute and chronic hepatitis, liver cirrhosis, and hepatocellular carcinoma (HCC). Worldwide, 2 billion people are infected with HBV, and more than 350 million are chronic carriers [1]. In addition, HBV infection causes more than 600,000 deaths per year [2]. So far, nucleoside analogues (NA) and alpha interferon (IFN-α) are the major therapies for HBV patients. However, long-term treatment with both NA and IFN-α may have drawbacks, including drug-resistant virus mutations and other side effects [3, 4]. Therefore, understanding of the molecular mechanism that determines HBV replication and pathogenesis is urgently needed.

The HBV genome is a partially double-stranded 3.2-kb DNA virus molecule that replicates via RNA intermediate [5]. Upon infection, relaxed circular DNA (rcDNA) is delivered into the nucleus through nuclear pore complexes after uncoating in the cytoplasm [6] and then is converted into a covalently closed circular DNA (cccDNA), which acts as the template for the transcription of viral RNAs (pgRNA and subgenomic RNA) [7]. HBV transcription is mainly controlled by four promoters (core, preS1, preS2, and X) and two enhancers (EN I and EN II). In addition, many of the host restriction factors regulate HBV by acting on the promoters and enhancers [8, 9].

Member of the zinc finger E-box binding homeobox (ZEB) family contain a similar structure of two zinc finger clusters and a central repression region, including CtBP and Smad-interacting domains. Zinc finger E-box binding homeobox 2 (ZEB2, also known as SIP1) was originally identified as a transcription factor and played vital roles in the TGF-β signaling cascade [10, 11]. In addition, ZEB2 is a Smad-interacting, DNA-binding, multi-zinc finger transcription factor [10, 12] involved in multiple cellular functions. Previous studies have documented that ZEB2 acts as a transcriptional repressor of E-cadherin and plays a role in epithelial-mesenchymal transition (EMT) in breast cancer and liver cancer [13, 14]. In contrast, ZEB2 has also been reported to function as a positive regulator of noncanonical Wnt signaling by transcriptionally upregulating vimentin [15, 16]. Intriguingly, Ellis et al. found that ZEB2 could bind to Zp via the ZV element and repress transcription initiated from Zp in Epstein-Barr virus (EBV) [17], which suggested a possible function of ZEB2 in modulating virus infection. However, the role of ZEB2 in HBV replication is unclear.

This study is the first to reveal an inhibitory effect of ZEB2 on HBV replication. ZEB2 may bind to HBV core promoter and inhibit its promoter activity, leading to inhibition of HBV DNA replicative intermediates, 3.5kb mRNA, core protein levels, and HBsAg and HBeAg secretion. Data from the present study indicated a negative regulatory role of ZEB2 in HBV replication and identified a novel host restriction factor in the HBV replication process.

## RESULTS

### Down-regulation of ZEB2 in HBV-expressing cells

To identify a potential relationship between HBV replication and the transcription factor ZEB2, the study first examined expression of ZEB2 in HepG2, HepG2.2.15—which is another HepG2 cell line that stably expresses HBV, and human hepatoma HepG2 cells transiently transfected with HBV expressing plasmid pCH-9/3091, which contains a 1.1-unit length HBV genome driven by CMV promoter.

**Figure 1:**
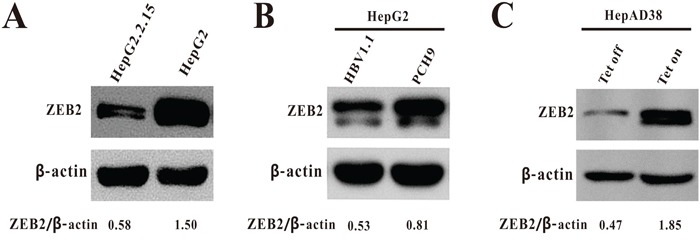
The expression of ZEB2 in HBV-expressing cells **A.** Expression of ZEB2 in HepG2.2.15 and HepG2 cells was analyzed using western blot. β-actin was used as a loading control. ZEB2/β-actin ratios were quantified by densitometric analysis with Quantity One software (Bio-Rad). Results presented were representative of 3 independent experiments. **B.** Expression of ZEB2 in HepG2 cells transiently transfected with plasmid pCH-9/3091 or vector pCH9 was analyzed using western blot. β-actin was used as a loading control. The ZEB2/β-actin ratio was quantified. **C.** Expression of ZEB2 in HepAD38 cells. Cells were cultured in medium with 0.3 μg/ml of tetracycline (Tet on: no HBV replication) and without tetracycline (Tet off: HBV replication).

The protein level of ZEB2 was downregulated in HBV-expressing cells relative to their control cells (Figure 1A, 1B). The present study further examined ZEB2 expression in the HepAD38 cell line, in which HBV replication can be regulated by tetracycline. The protein level of ZEB2 in the HepAD38 cells without tetracycline was much lower than in the HepAD38 cells with tetracycline (Figure 1C). Taken together, the results revealed that ZEB2 was significantly down-regulated by HBV replication, suggesting that transcription factor ZEB2 might play a role in HBV replication.

### ZEB2 overexpression inhibited HBV replication

**Figure 2:**
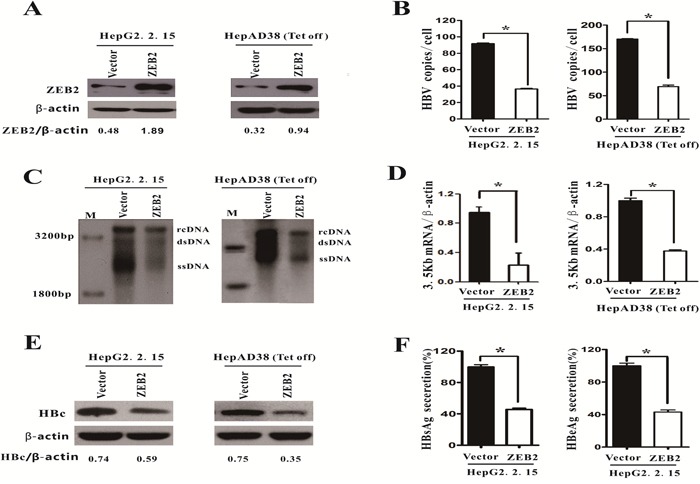
Overexpression of ZEB2 inhibited HBV replication HepG2.2.15 and HepAD38 cells were transfected with pcDNA4hismaxCZEB2 (ZEB2) or pcDNA4hismaxC (vector), respectively. **A.** Cells were collected 5 days after transfection, and ZEB2 expression was analyzed using western blot to evaluate transfection efficiency. ZEB2/β-actin ratios were quantified. Tet off: cells cultured without tetracycline. **B-C.** HBV intermediates were extracted and subjected to real-time PCR and southern blot analysis. rcDNA: intracellular HBV relax circle; dsDNA: double-stranded DNA; ssDNA: single-stranded DNA (* p < 0.01). **D.** The 3.5kb mRNA levels were determined by real-time PCR. **E**. Western blot analysis of HBV core protein expression in HepG2.2.15 and HepAD38 cells. β-actin was used as a loading control, and the HBc/β-actin ratio was quantified using Quantity One software (Bio-Rad). **F.** HBeAg and HBsAg levels in culture medium of HepG2.2.15 cells were collected and detected with ELISA. O.D. values were expressed as a percentage relative to the vector. All results shown as the means ± SD (* p < 0.01).

To elucidate the functional role of ZEB2 in HBV replication, HepG2.2.15 and HepAD38 cells were transfected with either pcDNA4his/maxC-ZEB2 (ZEB2) or pcDNA4his/maxC. The transfection efficiency of ZEB2 was confirmed by using western blot analysis (Figure 2A). Both real-time PCR (Figure 2B) and southern blot analysis (Figure 2C) revealed that ZEB2 overexpression significantly decreased HBV DNA replicative intermediates in the two HBV-expressing cell lines examined. In addition, ZEB2 overexpression inhibited HBV 3.5 kb mRNA expression. Furthermore, a reduction of more than 50% in 3.5 kb mRNA was observed in both types of cells (Figure 2D). Moreover, overexpression of ZEB2 resulted in a significant decrease in expression of core protein in HepG2.2.15 and HepAD38 cells (Figure 2E). ELISA assay was used to examine the HBeAg and HBsAg secretion in the culture medium of HepG2.2.15 cells. As expected, HBeAg and HBsAg secretion levels were decreased in HepG2.2.15 cells that overexpressed ZEB2 (Figure 2F). These results suggested that ZEB 2 could repress HBV replication.

### Silencing of ZEB2 promoted the replication of HBV

**Figure 3:**
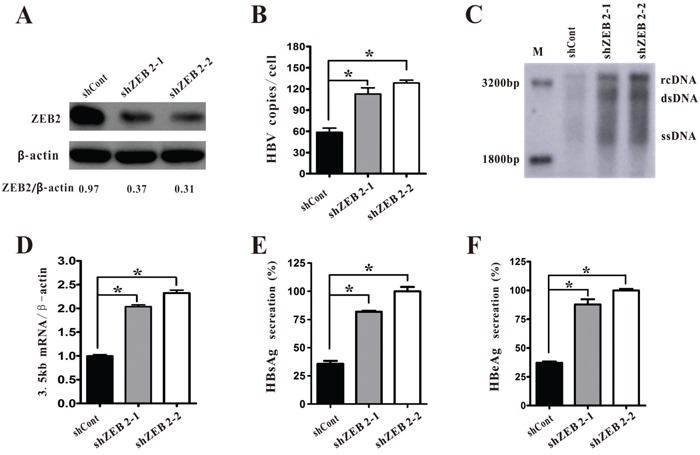
ZEB2 silencing promoted HBV replication in HepG2.2.15 cells **A.** HepG2.2.15 cells were infected with lentiviruses expressing shCont or ZEB2-targeting shZEB2-1 and shZEB2-2. Western blot analysis of ZEB2 was used to confirm the silencing efficiency, and ZEB2/β-actin ratios were quantified. **B-C.** HBV DNA replicative intermediates were determined in ZEB2 knockdown cells using real-time PCR (B) and southern blot analysis (C) (* p < 0.01). **D.** HBV 3.5kb RNA in HepG2 cells was analyzed by real-time PCR. **E-F.** HBsAg and HBeAg secreted in the supernatant were analyzed by ELISA assay. O.D. values were expressed as a percentage relative to the shZEB2-2. All results shown as the means ± SD (* p < 0.01).

To further elucidate the role of ZEB2 in HBV replication, the HepG2.2.15 cells were transduced with lentiviruses expressing scramble control (shCont) or ZEB2-targeting shRNAs (shZEB2-1 and shZEB2-2). The two independent shRNAs, shZEB2-1 and shZEB2-2, showed strong ZEB2 knockdown effects in HepG2.2.15 cells (Figure 3A). Both real-time PCR and southern blot results revealed that knockdown of ZEB2 markedly increased the level of HBV DNA replicative intermediates (Figure 3B, 3C). In addition, HBV 3.5 kb mRNA was remarkably upregulated in ZEB2-depleted HepG2.2.15 cells (Figure 3D). Consistently, the secretions of HBsAg and HBeAg in supernatant were increased in ZEB2-silencing cells compared with the control cells (Figure 3E, 3F). Taken together, these data suggested that ZEB2 knockdown promotes the replication of HBV.

### ZEB2 inhibits HBV replication by attenuating activities of HBV core promoter via binding to CACCT

**Figure 4:**
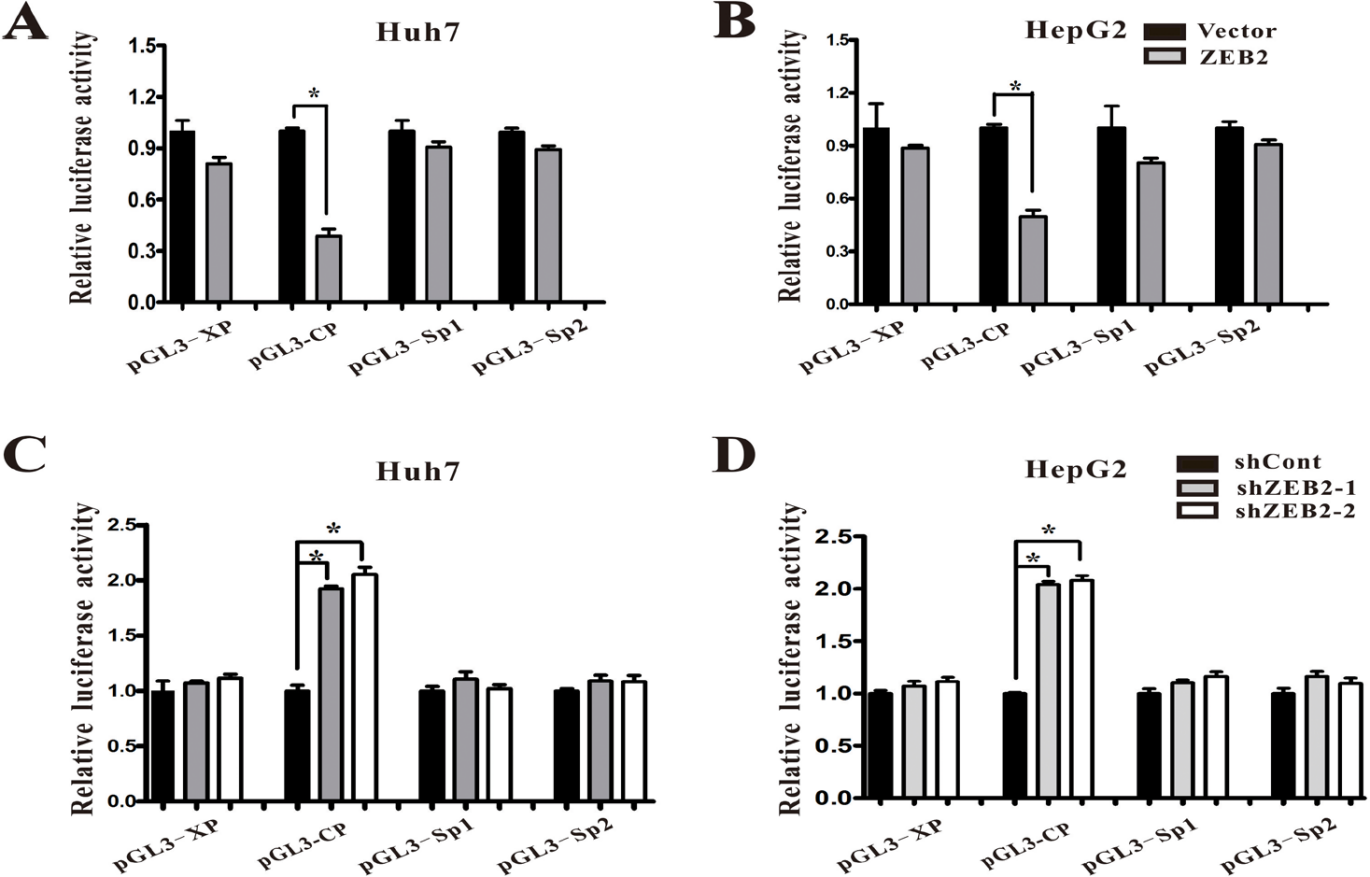
ZEB2 regulated HBV promoter activities **A-B.** Effect of ZEB2 overexpression on HBV promoters. Various luciferase reporter vectors were co-transfected with pcDNA3.1-ZEB2 into Huh-7 and HepG2 cells. Plasmid RL-TK was co-transfected to normalize transfection efficiency. Luciferase activity was measured 2 days after transfection. For each group, 3 independent experiments were conducted (* p < 0.001). **C-D.** Effect of ZEB2 knockdown on HBV promoters. Various luciferase reporter vectors were co-transfected with lentiviruses expressing shCont or ZEB2-targeting shZEB2-1 or shZEB2-2 into Huh-7 (C) and HepG2 cells (D). Plasmid RL-TK was co-transfected to normalize transfection efficiency. Luciferase activity was measured 3 days after transfection. All results shown as the means ± SD (* p < 0.001).

To further characterize the mechanism by which ZEB2 acted on HBV replication, the study used dual luciferase reporter assays to identify the role of ZEB2 in the activities of the HBV core, X, Sp1, and Sp2 promoters. The results showed that ZEB2 overexpression in Huh-7 and HepG2 cells suppressed core promoter activity and had no effect on X, Sp1, and Sp2 promoters (Figure 4A, 4B). In contrast, ZEB2 suppression elevated core promoter activity in Huh-7 and HepG2 cells (Figure 4C, 4D). Ectopic expression of ZEB2 significantly suppressed the activity of HBV core promoter, whereas ZEB2 knockdown resulted in a recovery of luciferase activity driven by core promoter.

**Figure 5:**
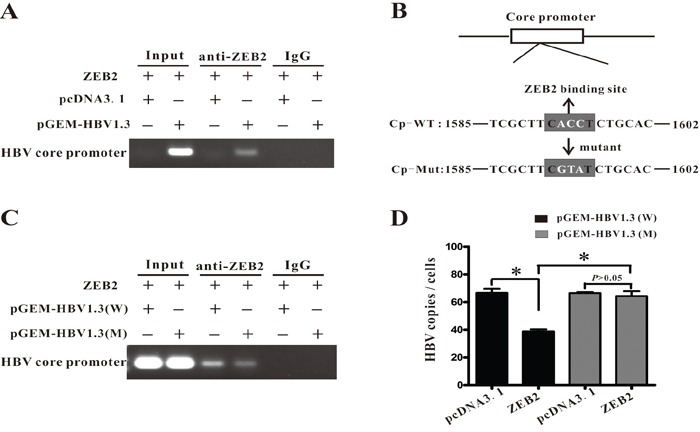
ZEB2 regulated HBV replication by interacting with HBV core promoter **A.** ChIP assay was performed to confirm the interaction between ZEB2 and HBV core promoter. Plasmid ZEB2 was co-transfected with pGEM-HBV1.3 into Huh7 cells. IgG of mouse was used as the control group. Input: The sample of DNA from total cell extract. **B.** The ZEB2 binding site (5′-CACCT-3′) was found in the HBV core promoter region. Point mutation of the binding site is in white (ACC→GTA). Cp-WT: the sequence of wild type HBV core promoter. Cp-Mut: the sequence of mutant ZEB2-binding site on HBV core promoter. **C.** Mutation of ZEB2 binding site decreased binding activity of ZEB2 to HBV core promoter. HepG2 cells were co-transfected with vector expressing ZEB2 and plasmid pGEM-HBV1.3 or pGEM-HBV1.3 mut (containing mutated ZEB2 binding site). ChIP assay was conducted 3 days after transfection. **D.** Mutated ZEB2 binding site eradicated ZEB2-induced inhibition of HBV replication. HepG2 cells were co-transfected with vector expressing ZEB2 and plasmid pGEM-HBV1.3 or pGEM-HBV1.3 mut. Effect of ZEB2 on HBV replication was analyzed by real-time PCR (* p < 0.01).

ZEB2 has been reported to bind to 5′-CACCT sequences in various promoters, including Xenopus brachyury promoter [18]_._ The present study scanned the sequence, revealing an E-box of CACCT within the HBV core promoter. Then, the study conducted a chromatin immunoprecipitation (ChIP) assay to investigate whether ZEB2 could bind to this sequence in HBV core promoter. Fragments of HBV core promoter were detected in anti-ZEB2 antibody immuno-precipitated candidates in Huh-7 cells transfected with pGEM-HBV1.3, but were not detected in cells transfected with control vector (Figure 5A). Then, the study mutated the ZEB2-binding site on the HBV core promoter in plasmid pGEM-HBV1.3 (Figure 5B). As expected, this mutation decreased the ability of ZEB2 to bind to the HBV core promoter (Figure 5C). Importantly, the ZEB2 binding-site mutation also eradicated the inhibition of HBV replication that was induced by ZEB2, as evidenced by real-time PCR analysis (Figure 5D). Taken together, these data demonstrated that ZEB2 can bind to core promoter and regulate HBV replication.

## DISCUSSION

As a transcription factor, ZEB2 is widely known to be involved in tumor progression and aggressiveness [19–21], embryogenesis development [22], and Mowat Wilson syndrome (MWS) phenotypes [23]. However, little is known about its roles in infectious diseases. Previous studies have discovered that ZEBs might play a role in regulating the EBV lifecycle. Janet E. Mertz et al. [24] has reported that both ZEB1 and ZEB2 can control the EBV latent-lytic switch by decreasing the expression of BZLF1, a virus-coded, multifunctional, DNA-binding protein belongs to the bZIP family of transcription factors. ZEBs bind to the sequences of 5′-CAGGTA-3′ (ZV) and 5′-CACCT-3′ (ZV') within the BZLF1 promoter and then repress their transcription, predisposing a cell toward establishing a latent state of infection. In addition, Lacher et al. have demonstrated that ZEB1/ZEB2 can bind to E2 box1 (CACCTG) in coxsackie virus and adenovirus receptor promoter, limiting adenoviral infectability by transcriptionally repressing it [25].

Based on these discoveries in EBV and the coxsackie virus, we speculated on a possible role of ZEB2 in regulating HBV replication. Results of the present study suggested a negative influence of ZEB2 on HBV transcription and replication. Overexpression of ZEB2 decreased HBV transcription, replication, and protein expression, whereas depletion of ZEB2 via shRNA upregulated HBV replication and expression. Luciferase activity analysis and ChIP assays further confirmed the role of ZEB2 in HBV replication. ChIP assays indicated that ZEB2 bound to core promoter via the CACCT sequence. When the E-box site in the HBV core promoter was mutated, binding of ZEB2 to the core promoter was significantly reduced, although not completely eradicated. Consistently, mutation of this binding site abrogated the inhibitory effect of overexpressed ZEB2 on HBV replication. Taken together, these results strongly indicated that ZEB2 plays an important role in negatively regulating HBV replication and expression by binding to core promoter.

Undoubtedly, members of host transcriptional factors in addition to ZEB2 contribute to regulating HBV replication and expression by affecting the activities of its promoters and enhancers. For example, liver-enriched hepatocyte nuclear factor (HNF) has been reported to stimulate HBV transcription and replication by binding to HBV promoters and enhancers [26]. TGF-β has been reported to suppress HBV replication through TGF-β/BMP signaling [27]. Our group has also reported that host factors such as SIRT1 and Cyclin D2 promote HBV replication [28, 29]. During the HBV lifecycle, transcriptional factors may function through direct or indirect pathways, involving some other molecules as co-activators or co-repressors. The present study observed a direct binding of ZEB2 to HBV core promoter, which may result in a direct inhibitory effect of transcription. However, other factors that continue to be elusive may also be involved in the process.

Interestingly, this study observed that ZEB2 decreased in HBV-expressing cells. Shinozaki et al. discovered that EBV upregulated ZEB1 and ZEB2 expression through downregulation of the miR-200 family, which targets ZEBs and further help establish a stable latent infection [30]. This study speculated that HBV may use some unknown strategies, such as DNA methylation, histone acetylation, other transcription factors, or microRNA, to antagonize the attack of ZEB2 and then facilitate its survival in the process of infection. However, more studies are needed to clarify this mechanism.

Briefly, this study identified a host transcriptional factor, ZEB2, as a novel repressor in HBV replication and expression by regulating its core promoter activity. The study provided information important to understanding the interaction between host factors and HBV, which might shed new light on viral pathogenesis or antiviral strategies. Further studies should be conducted to elaborate details of the mechanism of ZEB2-mediated HBV suppression and to assess its role in the actual infection process.

## MATERIALS AND METHODS

### Plasmids and antibodies

The plasmid ZEB2 (pcDNA4his/maxC-ZEB2) was constructed by ligating two fragments approximately 1.8 kb in length that included the entire ZEB2-coding region into pcDNA4hismaxC, which was a gift from professor Janet E. Mertz (McArdle Laboratory for Cancer Research; USA) [17]. ZEB2 shRNA (ZEB2 targeting) or nontargeting shRNA (shCont) was cloned into a lentivirus plasmid vector, plentilox-3.7. The sequence of ZEB2 targeting shRNA is 5′-CGGACCTTATGGCTACAGTAAC-3′ and 5′-CTCAGAGTCCAATGCAGCACTTAGGTGTA-3′. The sequence of shCont is 5′-GCACTACCAGAGCTAACTCAGATAG TACT-3′. The construction of the recombinant plasmid and package of lentivirus followed the method described previously [31]. Plasmid pCH-9/3091 was provided by Prof. Lin Lan (The Third Military Medical University; China). The pGEM-HBV1.3 (WT) was a gift from U. Protzer (University of Heidelberg; Germany). Rabbit anti-ZEB2 polyclonal antibody (ab25837) was obtained from Abcam (USA), and HBV core antibody from Dako (B0586; USA).

### Point mutation of HBV1.3

The pGEM-HBV1.3 (Mut) was mutated at the binding site of ZEB2 with PCR. In brief, the HBV sequence was amplified from pGEM-HBV1.3 by PCR using the sense primer P1 (5′-cggaccgtgtgcacttcgcttcgtatctgcacgtagcatggagaccaccg-3′) and the antisense primer P2 (5′-cggtggtctccatgctacgtgcagatacgaagcgaagtgcacacggtccg-3′). The sense primer and the antisense primer were reverse compliment, and the primer contained the mutated ZEB2 binding sites. Then, the PCR product was purified and digested with DpnI (Promega; USA). After digestion, the mutated pGEM-HBV1.3 was obtained by electro-transformation and extracted by Plasmid Mini Kit (OMEGA; USA). Finally, the mutated pGEM-HBV1.3 was identified by DNA sequencing.

### Cell culture and transfection

HepG2 and Huh7 were maintained in modified eagle medium (MEM), supplemented with 10% fetal-calf serum, 100 U/ml of penicillin, and 100 U/ml of streptomycin. HepAD38 cells (in which HBV replication can be regulated by tetracycline) [32] were cultured in MEM with 10% fetal-calf serum. HepG2.2.15 cells (stably HBV-transfected HepG2 cells, as well as expression of virus-related proteins) were cultured in MEM containing 10% fetal-calf serum and 500 μg of G418/ml. All cells were incubated at 37°C under 5% CO_2_ in a humidified incubator. Transfection was carried out using Lipofectamine 2000 (Invitrogen; USA), and cells were collected 5 days after transfection.

### Lentivirus production

Lentivirus that expressed shZEB2-1, shZEB2-2, or shCont was produced in HEK-293FT cells using the pLentilox-3.7 vector expressing corresponding shRNA with the aid of packaging plasmids pLP1, pLP2, and pLP/VSVG from the BLOCK-iT Lentiviral RNAi Expression System (Invitrogen; Carlsbad, CA). Viruses were concentrated by using PEG-it virus precipitation solution (System Biosciences; Mountain View, CA) according to instructions and stored at −80°C.

### Southern blot analysis

Extraction of HBV replicative intermediates was performed as described by Zhenzhen Zhang et al. and Jihua Ren et al. [28, 33]. Briefly, DNA samples were separated on 0.9% agarose gels and transferred onto nylon membranes (Roche; Germany). After UV cross-linking and prehybridization, the membrane was hybridized with a digoxigenin-labeled HBV-specific probe generated by using a Random primed labeling kit (Roche; Germany) and then exposed to X-ray film to detect the signals.

### Protein extraction and western blot

Protein was extracted from cells according to instructions in a Protein Extract Kit (KaiJi; China), and protein content was measured with a BCA protein assay (Bio-Rad). Total proteins (30 μg) were separated by SDS-PAGE and transferred to a nitrocellulose membrane. After the membranes were incubated with primary antibodies (anti-SIP1, 1:1,000; anti-core protein, 1:1,000; anti-actin, 1:2,000) overnight at 4°C and subsequently incubated with Horseradish peroxidase (HRP)-conjugated secondary antibodies at room temperature for 1h, blots were developed with enhanced chemiluminescence reagents (Pierce; USA).

### Quantitative real-time PCR

Total cellular RNAs in cells were isolated with Trizol reagent (Invitrogen, USA) according to the manufacturer's protocol. Reverse transcription was conducted with a PrimeScript RT Reagent Kit with gDNA Eraser (Takara; Japan). Real-time quantification PCRs of HBV replicative intermediates and 3.5Kb mRNA were performed using SYBR Green Master (Roche; Germany). The primers used for 3.5 kb mRNA were 5′-GCCTTAGAGTCTCCTGAGCA-3′ (forward) and 5′- GAGGG AGTTCTTCTTCTAGG-3′ (reverse). HBV specific primers were 5′-CCTAGTAG TC AGTTATGTCAAC-3′ (forward), and 5′-TCTATAAGCTGGAGTGCGA-3′ (reverse). All expression values of target genes were calculated using the 2−DCt method.

### Detection of HBsAg and HBeAg

The levels of HBV surface antigen (HBsAg) and HBV e-antigen (HBeAg) in culture medium were determined according to the manufacturer's instructions using an enzyme-linked immunosorbent assay (ELISA) (KHB; Shanghai, China). Each experiment was performed at least three times.

### Luciferase assay

Luciferase activity in cell lysates was detected with a dual luciferase reporter assay system (Promega; USA). Cells were collected after co-transfecting luciferase report vectors (pGL3-Cp, pGL3-Xp, pGL3-Sp1, and pGL3-Sp2) with either pcDNA4hismaxC-ZEB2, shRNA, or control vector for 36 h, and luciferase activity was detected in the lysates using a GloMax microplate luminometer (Promega; USA). Luciferase activity was normalized by co-transfecting with pRL-TK (Promega).

### Chromatin immunoprecipitation assay

Cells were co-transfected with either pcDNA4hismaxC-ZEB2, pGEM-HBV1.3 (WT), pGEM-HBV1.3 (Mut), or control vector and fixed with 1% formaldehyde for 10 min at 37°C. Cells were resuspended and sonicated into small fragments. The supernatants were subjected to immunoprecipitation and incubated with relative antibodies, including anti-ZEB2. The immunoprecipitated DNA was amplified by PCR with HBV core promoter-specific primers: 5′-GCACTTCGCTTCACCTCTGCACGTAGCAT G -3′ (forward) and 5′- AACCTAATCTCCTCCCCCAACTCCTCCCAG-3′ (reverse).

### Statistical analysis

Student t-tests and Fisher's exact tests was used to access the differences between groups. A p value of < 0.05 was considered as statistically significant. All statistical analyses were performed using SPSS16.0.
